# The Mulli Hypothesis: an ethnobiological framework for studying the phytopharmaceutical potential of *Schinus molle* (Anacardiaceae)

**DOI:** 10.3389/fphar.2026.1939043

**Published:** 2026-09-04

**Authors:** Álvaro Puelles-Díaz, Mercedes Di Pasquo, Claudia Bernal Zuluaga

**Affiliations:** 1 Facultad de Ciencias, Universidad de La Serena, La Serena, Chile; 2 Carrera de Medicina, Universidad del Alba, La Serena, Chile; 3 Laboratorio de Palinoestratigrafía y Paleobotánica, Centro de Investigaciones Científicas y Transferencia de Tecnología, CICYTTP-CONICET, Entre Ríos, Argentina; 4 Laboratorio de Catálisis y Biocatálisis, Departamento de Química, Facultad de Ciencias, Universidad de La Serena, La Serena, Chile

**Keywords:** Anacardiaceae, Andean ethnobotany, chemotype, ethnopharmacological unit, ethnopharmacology, phytopharmaceutical standardization, Schinus areira, Schinus molle

## Abstract

**Background:**

*Schinus* *molle* L. (Anacardiaceae), the Andean “molle” or “mulli,” is among the longest-used trees of western South America. Despite more than 3 decades of pharmacological research, reports remain heterogeneous and no reproducible phytopharmaceutical candidate has emerged—an inconsistency that may arise less from insufficient experimentation than from the biological object never having been adequately defined.

**Aim:**

This article integrates the historical-linguistic, archaeological, ethnobotanical and taxonomic record and proposes a falsifiable framework—the Mulli Hypothesis—for how the species should be studied. The aim is not to validate the hypothesis, but to establish why the evidence makes it a warranted and testable hypothesis.

**Argument:**

First, molle derives from Quechua mulli, a pre-Linnaean vernacular. Second, the most secure ancient evidence is large-scale chicha de molle at the Wari site of Cerro Baúl, corroborated by molle-specific triterpenoid biomarkers. Third, Andean ethnomedicine consistently records external analgesic and anti-inflammatory use of the leaf; modern pharmacology has begun to report activities consistent with this specific observation while also reporting activities the tradition never described. Fourth, nomenclatural instability means the identity of “mulli” is itself unstable, and reported variability may be partly a species-identity artefact and partly a genuine chemotype signal.

**Proposal:**

Historical knowledge appears to preserve a reproducible pharmacological signal for a heterogeneous biocultural entity. We propose the ethnopharmacological unit as an operational, falsifiable model underpinning a framework for future standardization. Although developed for molle, the ethnopharmacological unit is proposed as a potentially generalizable analytical approach for investigating irreproducibility where a botanical name may conceal biological, chemical or cultural heterogeneity.

## 1 Introduction

Despite more than 3 decades of pharmacological research on Schinus molle, no reproducible phytopharmaceutical candidate has emerged. Rather than reflecting insufficient experimentation, this inconsistency may arise because the biological object itself has never been adequately defined. Irreproducibility of this kind is now recognized as a systemic challenge in natural-product pharmacology, where variation in botanical identity, chemical composition and preparation routinely frustrates replication.

Few South American trees carry as long a human relationship as the Peruvian pepper tree, Schinus molle L. (Anacardiaceae). Native to the western Andean slope from Peru through Bolivia and northern Chile to central Argentina, it has supplied food, drink, medicine, dye, resin and ritual material for more than a millennium and remains central to Andean traditional medicine. A substantial modern literature, including genus-level reviews, reports antimicrobial, antioxidant, insecticidal, anti-inflammatory and antinociceptive activities for its extracts and essential oil (Barrachina et al., 1997; Bello et al., 1998; Bendaoud et al., 2010; El-Nashar et al., 2022). Yet these reports vary widely, taxonomic identity is contested, and chemistry shifts with geography, organ, harvest season and extraction method (Radice et al., 2026). This variability is not a peripheral problem: it challenges the assumption that the Latin binomial alone is a sufficient unit for interpreting the pharmacology of “molle.”

This study asks how the historical and ethnobotanical knowledge of *Schinus molle* has shaped, and how it should discipline, modern pharmacological research. We argue that averaging the variability away is the error, and that heterogeneity is the object worth studying. We do not claim that history explains the whole pharmacology. More cautiously, historical and ethnomedical knowledge preserves a specific pharmacological signal, above all the external anti-inflammatory and analgesic use of the leaf, within an entity, “molle,” that may not be biologically, chemically or culturally singular.

The point is not merely botanical. “Mulli” belongs to a long Andean biocultural field in which plants are ingredients of subsistence, social exchange, healing, memory and place-based practice. The use of molle in brewing, ritual and medicine therefore cannot be separated from local ecologies, local names, plant parts and preparation practices. In ethnopharmacology, this matters because it shifts the unit of analysis from an abstract Latin binomial to a historically situated relationship among people, plants and pharmacological expectations.

The aim of this study is not to validate the Mulli Hypothesis, but to establish why historical, ethnobotanical, taxonomic and phytochemical evidence make such a hypothesis warranted and testable. The empirical tests belong to subsequent studies on chemotypes, mechanisms and Chilean populations. Here, we provide the conceptual ground on which those studies by defining the Mulli Hypothesis, deriving its falsifiable predictions and proposing the ethnopharmacological unit as an operational model for future research.

## 2 The Mulli Hypothesis, its predictions, and the ethnopharmacological unit

**FIGURE 1:**
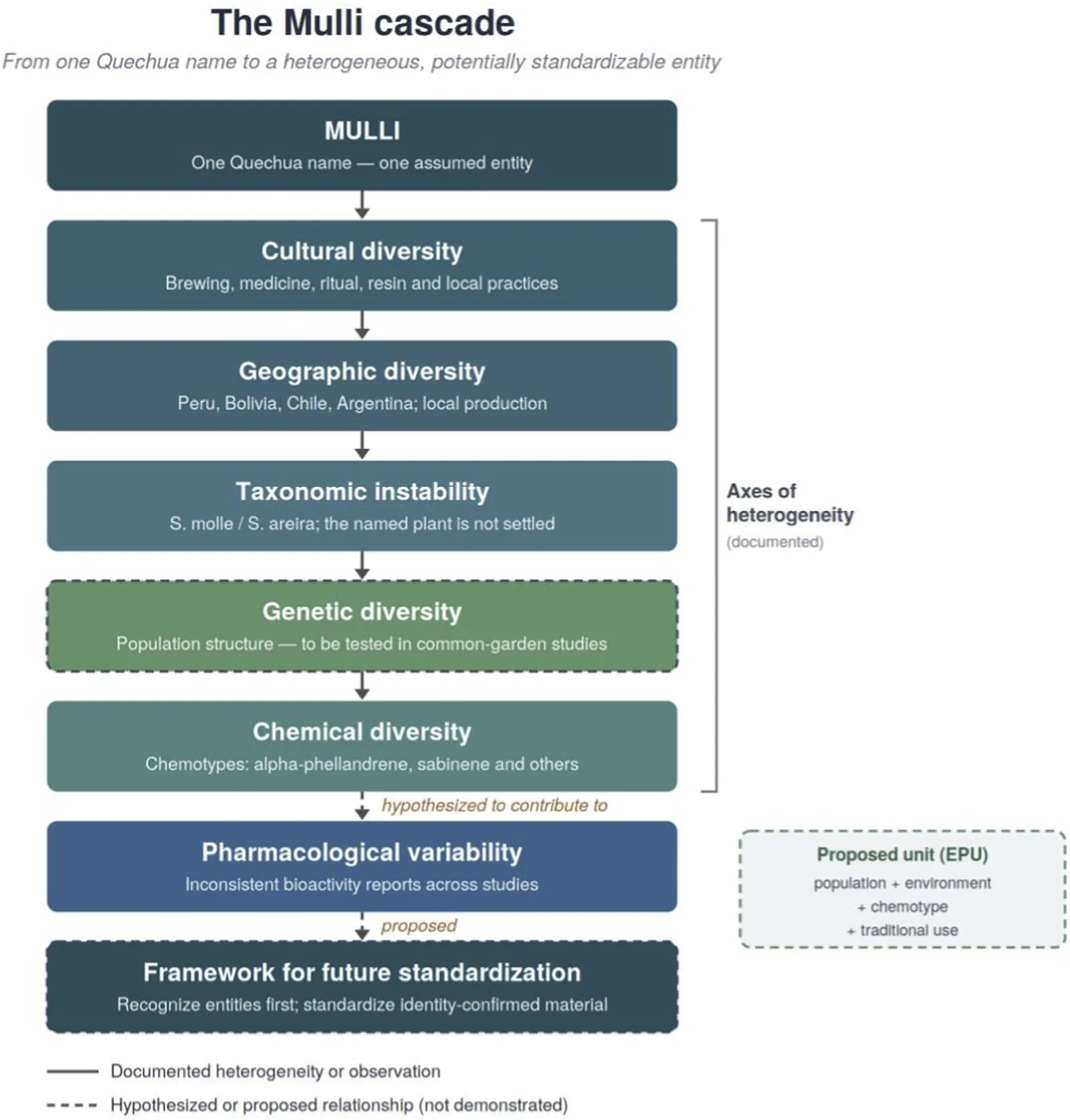
The Mulli cascade. From a single Quechua name to a heterogeneous, potentially standardizable entity: successive axes of heterogeneity that may frame the pharmacological variability reported for the species. Solid arrows denote documented axes of heterogeneity; the dashed arrow marks the hypothesized (not demonstrated) contribution to pharmacological variability.

Figure 1 renders the argument as a cascade: a single Quechua name overlying successive, partly independent axes of heterogeneity. The figure should not be read as a demonstrated causal sequence, but as a conceptual map of variables that must be made explicit before pharmacological variability can be interpreted or any future standardization attempted.

### 2.1 Predictions of the Mulli Hypothesis

A conceptual framework derives its scientific value from being falsifiable. The Mulli Hypothesis therefore yields explicit, testable predictions, each corresponding to a different level of evidence to be addressed across the series.

- P1. Distinct historical or geographic populations of “molle” are expected to show distinguishable gas chromatography–mass spectrometry chemotype profiles.
- P2. Chemotype is expected to predict anti-inflammatory and antinociceptive activity more consistently than provenance or species label alone.
- P3. If chemotype variation is not purely environmental, a meaningful share of that variation should persist under common-garden conditions, indicating partial heritability.
- P4. An integrated ethnopharmacological-unit classification should explain pharmacological variance better than conventional taxonomic classification alone.
- P5. If traditional practice has tracked biologically meaningful variation, traditional-use clusters should align more closely with chemotype or ethnopharmacological-unit clusters than with nominal species labels alone.

Any of these can fail. If chemotype neither tracks geography nor predicts activity, if all variation collapses under common-garden conditions, or if traditional-use clusters do not align with chemical or pharmacological groupings, the hypothesis would be falsified or reduced to environmental plasticity. Such an outcome would be scientifically useful and should be reported as readily as the converse.

To make these predictions operational rather than narrative, Table 1 specifies for each one the variables, a study design, a statistical approach, and an explicit criterion for confirmation or falsification. Each prediction is thereby stated as a hypothesis that a defined study could reject. For the model comparisons in P2 and P4 in particular (Box 1), the ethnopharmacological-unit model must not be judged superior merely because it carries more variables than the species-label model; the designs therefore rely on nested model comparison, complexity-penalized fit (AIC/BIC) and cross-validated assessment of incremental predictive value, together with *a priori* sample-size and effect-size targets and control of known confounders.

**TABLE 1:** Operationalization of the predictions of the Mulli Hypothesis.

Prediction	Variables	Study design	Statistical approach	Confirmation/falsification
P1 — Whether populations differ in chemotype	Independent: source population (geographic/historical origin). Dependent: GC-MS chemotype profile	Voucher-confirmed accessions from multiple populations across the range; replicated GC-MS profiling	PCA/hierarchical clustering + PERMANOVA on chemical distance, population as factor	Confirmed: populations form significantly distinguishable chemotype clusters (PERMANOVA p < 0.05). Falsified: no chemical structure by population
P2 — Whether chemotype predicts activity better than label	Independent: chemotype vs. species label vs. provenance. Dependent: anti-inflammatory/antinociceptive activity (standardized assay)	Bioassay of chemotyped samples under a common protocol; competing-predictor comparison	Regression/mixed models; model-fit comparison (AIC, cross-validated accuracy) across predictor sets	Confirmed: chemotype explains significantly more activity variance than label or provenance. Falsified: chemotype adds no predictive value over label
P3 — Whether chemotype is partly heritable	Independent: growing environment (common garden vs. source); population. Dependent: chemotype profile	Common-garden trial of multiple populations grown under identical conditions	Variance partitioning/mixed model; heritable vs. environmental component	Confirmed: significant between-population chemotype variance persists under common garden. Falsified: differences collapse (variation is environmental)
P4 — Whether the EPU explains variance better than taxonomy	Independent: classification scheme (EPU vs. taxonomic label). Dependent: pharmacological outcome	Dataset with both EPU assignment and taxonomic label; outcome modelled under each scheme	Nested model comparison; variance explained (R^2^, likelihood-ratio test)	Confirmed: EPU explains significantly more pharmacological variance than label alone. Falsified: no improvement over taxonomy
P5 — Whether traditional use tracks chemistry rather than names	Traditional-use clusters vs. chemotype/EPU clusters vs. species-label clusters	Encode traditional uses (indication, part, preparation) across populations; cluster and test congruence	Cluster congruence (Mantel test/cophenetic correlation)	Confirmed: traditional-use clusters align more with chemotype/EPU than with species labels. Falsified: no better alignment than with labels

BOX 1 **The Mulli Hypothesis.**

The historical, taxonomic and phytochemical heterogeneity of *Schinus* suggests that “molle” may not correspond to a single, homogeneous pharmacological object. Instead, it may comprise geographically structured entities—chemotypes and, less certainly, deeper taxonomic lineages—whose conflation under a single name could contribute to the inconsistent pharmacological evidence reported for the species. If this is correct, recognizing and characterizing these entities would be a necessary first step toward any framework for the future standardization of a candidate phytomedicine.

Status. This is a hypothesis, not a finding. No genetic evidence yet establishes distinct entities, and no study has shown that chemotypes differ in pharmacological activity. It is advanced as a falsifiable framework, through predictions P1–P5, to be tested across the series.

**FIGURE 2:**
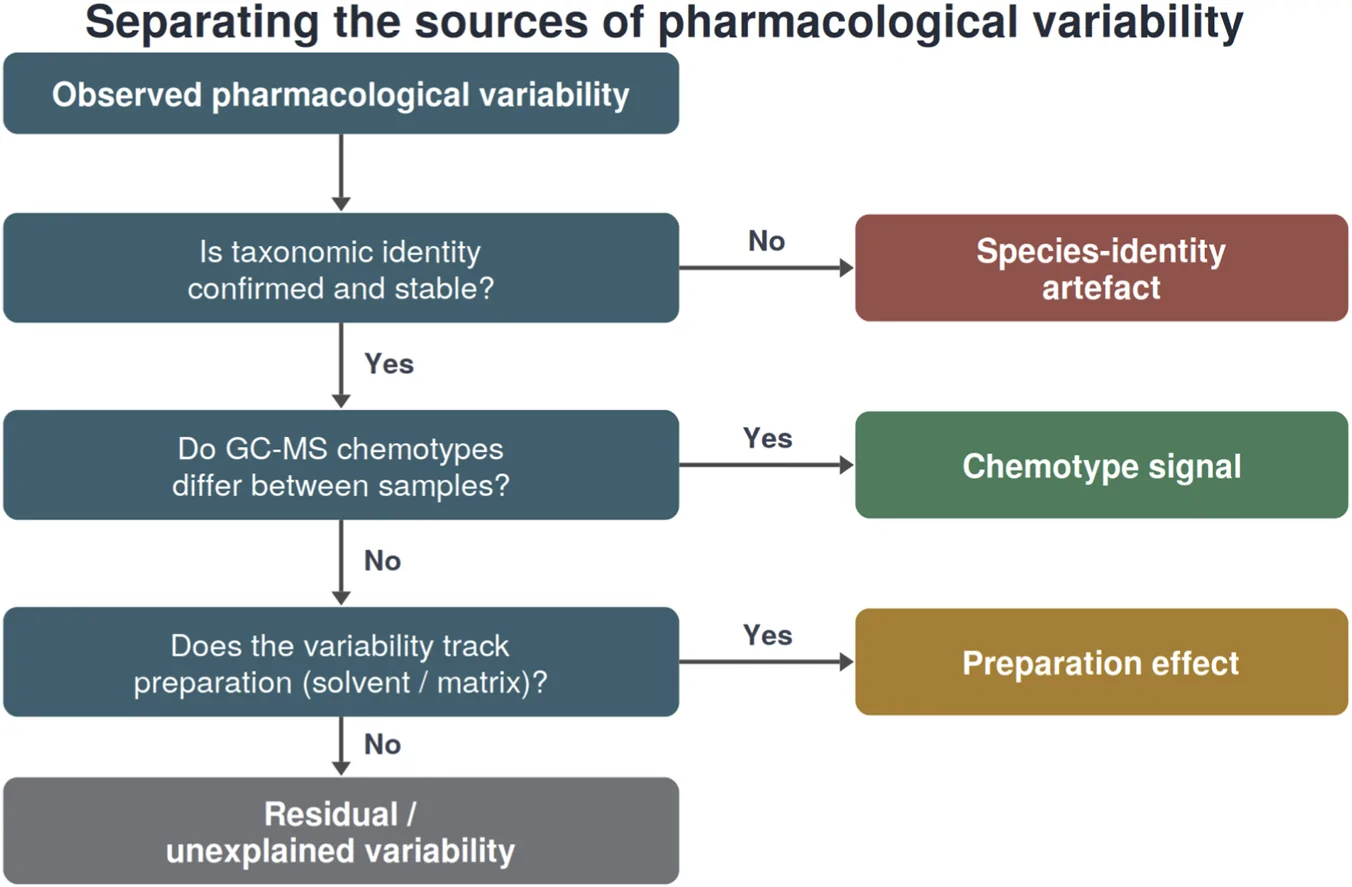
Separating the sources of pharmacological variability. A decision procedure distinguishing a species-identity artefact, a chemotype signal and a preparation effect as competing, testable explanations for the reported variability of molle.

These predictions also require separating three non-exclusive sources of the observed pharmacological variability, which are often conflated: a *species-identity artefact* (variation reflecting misidentification or unresolved taxonomic instability rather than biology), a *chemotype signal* (genuine intraspecific chemical variation), and a *preparation effect* (variation introduced by solvent, matrix or method). Figure 2 sets out a decision procedure that separates them: variability that disappears once taxonomic identity is confirmed is an identity artefact; variability that persists under confirmed identity and tracks the GC-MS profile is a chemotype signal; and variability that tracks preparation rather than chemistry is a preparation effect. This separation is itself testable, and each branch implies a different experimental follow-up.

### 2.2 The ethnopharmacological unit: an operational model

**FIGURE 3:**
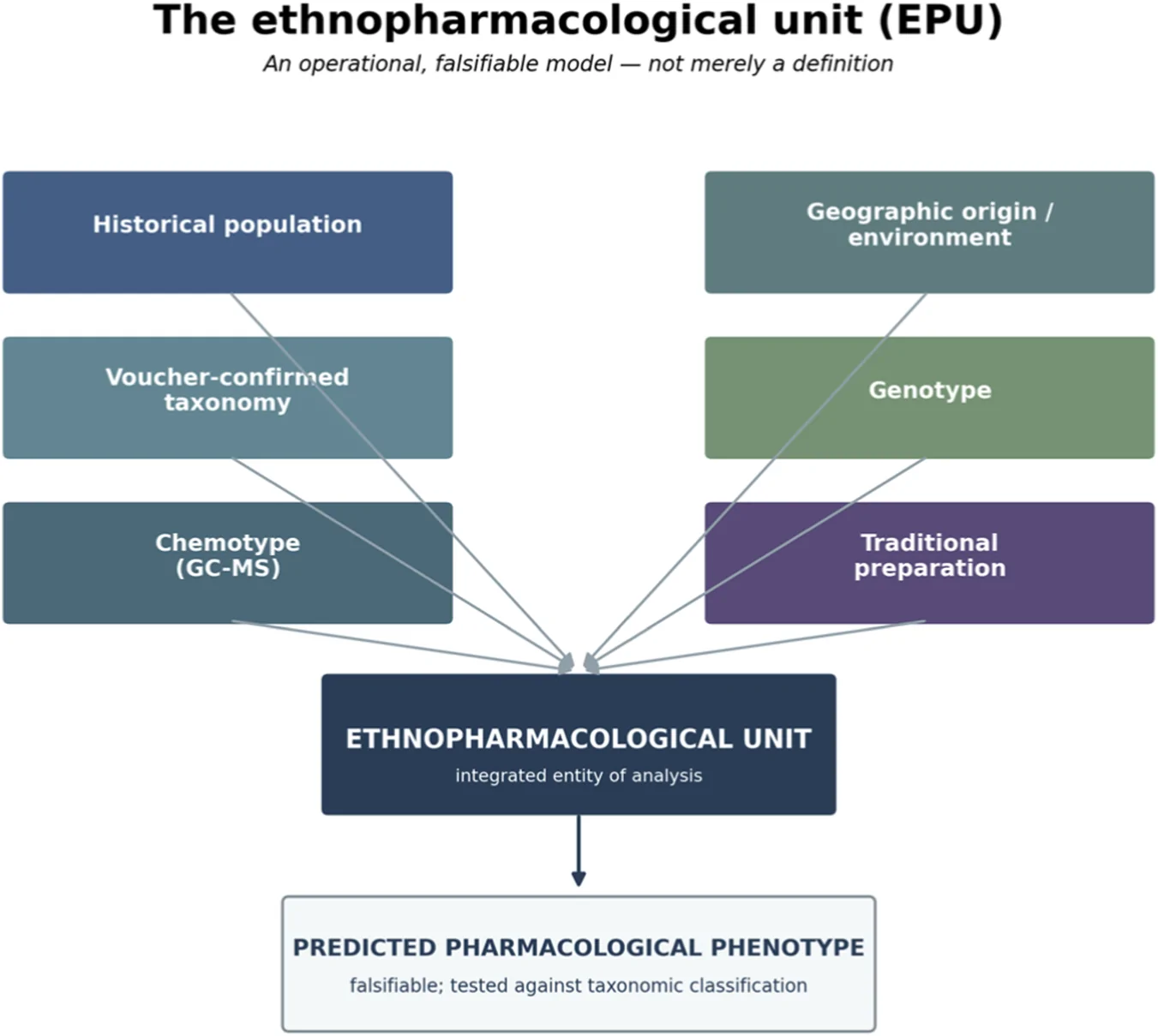
The ethnopharmacological unit (EPU): integrating the determinants of pharmacological phenotype. An operational, falsifiable model in which provenance, taxonomy, genotype, chemotype and traditional preparation jointly define a testable pharmacological expectation.

The Mulli Hypothesis provides the conceptual rationale, whereas the ethnopharmacological unit constitutes its operational implementation. To make the framework usable, we propose that the unit of study should not be the species alone but the ethnopharmacological unit: the integrated entity formed by a historical population, its geographic origin and environment, voucher-confirmed taxonomy, genotype, chemotype and traditional preparation, which together define a testable pharmacological expectation (Figure 3). The ethnopharmacological unit is intended not as a label but as an operational model with predictive content: it proposes that these covariates, taken together, may explain bioactivity more reliably than the Latin binomial alone. This model does not assume that all components contribute equally in every case. Rather, it requires that provenance, taxonomic identity, chemical profile and preparation be made explicit before pharmacological variability is interpreted.

Although developed here for *Schinus molle*, the ethnopharmacological unit is intended as a general framework for medicinal plants in which biological, chemical and cultural variability coexist. Similar challenges have been described in geographically structured medicinal taxa such as *Arnica montana*, *Curcuma longa*, *Cannabis sativa* and *Hypericum perforatum*, where taxonomic identity alone inadequately predicts phytochemical composition or therapeutic activity.

If validated, this unit need not be specific to *S. molle*. It could offer a general template for medicinal plants with high geographic variability and long, spatially structured traditions of use. That generalizability is flagged as a prospect, not a claim.

### 2.3 Operational definition and assignment of the ethnopharmacological unit

To move the ethnopharmacological unit from a conceptual proposal to a usable analytical tool, we define here the information it requires, the procedure by which it is assigned, and the confidence that can be attached to each assignment. The intention is that any investigator should be able to construct an EPU for any medicinal-plant record by following a reproducible procedure, and that two investigators working from the same primary source should arrive at the same EPU and the same confidence level.

An EPU is specified by a set of fields organised into three tiers (Table 2). *Mandatory* fields are those without which a pharmacological result cannot be attributed to a defined object: voucher specimen, accepted taxonomy (name, authority and family, validated against an accepted nomenclatural source), geographic origin, plant organ, preparation (extraction solvent or matrix and method), route of administration, and the traditional indication under examination. One field is *conditional*: the nomenclatural synonym history is required whenever the accepted name is taxonomically unstable—as it is for the *Schinus molle*/*S. areira* complex—and may be omitted where nomenclature is settled. *Optional* fields enrich the unit and raise its confidence but are not required for a minimal assignment.

**TABLE 2:** Fields defining an ethnopharmacological unit.

Field	Tier
Voucher specimen (herbarium accession)	Mandatory
Accepted taxonomy (name + authority + family)	Mandatory
Geographic origin (population/locality)	Mandatory
Plant organ	Mandatory
Preparation (solvent/matrix + method)	Mandatory
Route of administration	Mandatory
Traditional indication	Mandatory
Nomenclatural synonym history	Conditional (required when the name is taxonomically unstable)
Chemotype (GC-MS or equivalent profile)	Optional
Major metabolites	Optional
Archaeological evidence	Optional
Historical-linguistic evidence	Optional

Because primary sources vary in completeness, each field is additionally tagged for certainty as confirmed (directly documented in the source), inferred (reasonably deducible from the source but not stated), or unknown (not recoverable from the source). This tagging is what allows a heterogeneous literature to be compared without discarding it: a study lacking a voucher is not excluded but recorded as “voucher: unknown,” which itself becomes analysable information.

**FIGURE 4:**
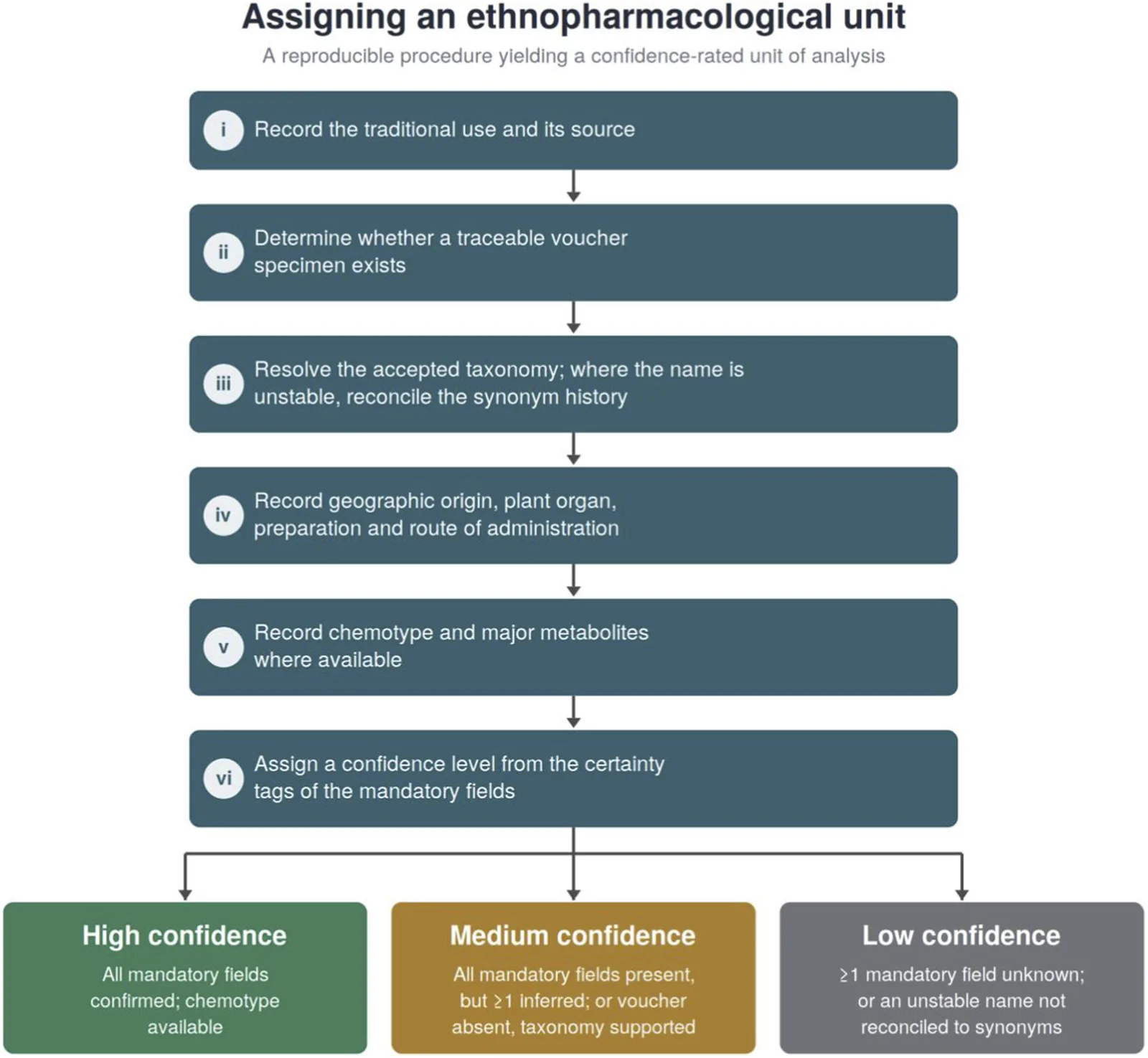
Assigning an ethnopharmacological unit. A reproducible procedure for constructing an EPU from a primary source and assigning a confidence level (high, medium or low) from the certainty of its mandatory fields.

Assignment proceeds in a fixed order (Figure 4): (i) record the traditional use and its source; (ii) determine whether a traceable voucher specimen exists; (iii) resolve the accepted taxonomy and, where the name is unstable, reconcile the synonym history; (iv) record geographic origin, plant organ, preparation and route; (v) record chemotype and major metabolites where available; and (vi) assign a confidence level from the certainty tags of the mandatory fields.

Three levels summarise the documentation completeness of an assignment (Table 3). Documentation is complete when all mandatory fields are confirmed and a chemotype is available; partial when all mandatory fields are present but one or more is inferred rather than confirmed, or when a voucher is absent although taxonomy is otherwise well supported; and incomplete when at least one mandatory field is unknown, or when an unstable name has not been reconciled against its synonym history. These categories describe the documentation completeness and traceability of the proposed unit, not its pharmacological validity or reproducibility; they are author-defined and provisional, and their inter-rater reliability and their relationship to pharmacological reproducibility remain to be evaluated. They are also distinct from the reliability of the biological identification itself, which is carried by the certainty tags of the individual taxonomic and chemotype fields rather than by the completeness label; genotype and validated-chemotype information, where available, is recorded in those fields rather than subsumed into a single category.

**TABLE 3:** Documentation-completeness levels for an EPU assignment.

Level	Criteria
High	All mandatory fields confirmed; chemotype available
Medium	All mandatory fields present, but ≥1 inferred; or voucher absent while taxonomy is otherwise well supported
Low	≥1 mandatory field unknown; or an unstable name not reconciled against its synonym history

Defined in this way, the EPU is portable: the same fields, tags and procedure can be applied to any species in which biological, chemical and cultural variability coexist. We hypothesize that organising the *Schinus* literature into EPUs, rather than pooling it under a single binomial, may reduce the apparent irreproducibility of its reported activities; this is stated as a testable expectation in the Mulli Hypothesis predictions (Section 2.1), not as a demonstrated result.

### 2.4 The ethnopharmacological unit in relation to existing frameworks

The ethnopharmacological unit is not a new method of botanical authentication, chemical standardization, or reporting, and it is important to state precisely what it adds to the several well-established frameworks that already address parts of the problem. Its contribution is not at the level of any single component but at the level of analysis: it integrates provenance, voucher taxonomy, chemotype and preparation into one unit and binds that unit to a testable pharmacological expectation. We situate it below relative to the main existing approaches.

The chemotype (or chemovar) concept captures intraspecific chemical variation and is central to the argument developed here; the reported chemical variability of *Schinus molle* is, in part, chemotypic and varies among geographic populations (Gomes et al., 2013; Radice et al., 2026). However, a chemotype describes chemical variation in isolation: it does not, by itself, tie that variation to taxonomic identity, geographic provenance, plant part, or a documented traditional use. The EPU incorporates chemotype as one field among several, so that chemical variation is interpreted jointly with the other determinants rather than as a free-standing signal.

Botanical authentication—including voucher documentation and DNA barcoding—addresses the prior and essential question of whether the material studied is the species it is claimed to be (Bennett and Balick, 2014). This is necessary but not sufficient for the problem addressed here. Correct species identity does not guarantee a stable pharmacological phenotype when the correctly identified species is itself chemically and culturally heterogeneous. Authentication answers “is this the right species?”; the Mulli Hypothesis asks the further question of whether the species name, even when correct, predicts the phenotype. The EPU treats authentication as a mandatory field but does not stop there.

Pharmaceutical standardization, including the drug-extract ratio and pharmacopoeial specifications, operates downstream of the problem: it presupposes that the object has already been defined and seeks batch-to-batch consistency of a product. The EPU operates upstream, at the stage where the object of study is defined; the two are complementary, and standardization becomes more tractable once a well-specified unit is in place.

Finally, the field has developed consensus best-practice statements that overlap closely with our concerns: the consensus statement on ethnopharmacological field studies (Heinrich et al., 2018), the analysis of common challenges in phytopharmacological research (Heinrich et al., 2020), and, most directly, the ConPhyMP guidelines for characterizing and reporting medicinal-plant extracts (Heinrich et al., 2022). These are reporting and practice standards—checklists that specify what should be documented and how. The EPU is complementary to them rather than a substitute: where ConPhyMP specifies how the plant material and its chemistry should be reported, the EPU provides the analytical unit that such reporting populates, and adds two elements that a reporting checklist does not supply—an explicit link from the characterized material to a documented traditional use, and a falsifiable prediction attached to that link. In this sense the novelty of the EPU lies not in any of its individual fields, each of which draws on established practice, but in their integration into a single, confidence-rated, predictive unit of analysis.

To be explicit about what the unit is and is not: in practical terms it is an analytical unit—the object over which pharmacological comparisons are made—coupled to a confidence-assessment scheme for that object, and it can also serve as a structured reporting format. Its distinctive contribution is not any individual field, all of which exist in current best practice, but the decision it forces: what counts as the same, or a different, material for the purpose of comparing results. We do not present it as a validated method or a demonstrated scientific entity, but as a proposed unit of analysis whose usefulness is to be tested through the predictions of Section 2.1.

The same distinction places the unit relative to the field’s more recent methodological currents. Quality-oriented standards define the material without predicting its phenotype: DNA barcoding and molecular authentication establish identity, and good agricultural and collection practice (World Health Organization, 2003) governs how the starting material is grown, collected and handled. Discovery-oriented approaches, by contrast, operate downstream of a defined object: metabolomics-guided standardization characterizes the full chemical profile of a given preparation, and network or systems pharmacology maps the targets and mechanisms of its constituents (Noor et al., 2022). Each is powerful within its level, and the ethnopharmacological unit is not a substitute for any of them. Its role is to supply the level they all presuppose: a defined, confidence-rated object—provenance, taxonomy, chemotype and preparation bound to a documented use—without which authentication has nothing to authenticate a phenotype against, standardization has no stable target, and a network model risks being built on material the next study cannot reproduce.

## 3 The name “mulli”: etymology and referential instability

The specific epithet molle derives not from Latin mollis (“soft”) but from the Quechua plant name mulli. The pre-Linnaean genus name Molle was used before Linnaeus adopted *Schinus* in Species Plantarum (Linnaeus, 1753) and survives as a generic synonym, Molle Mill. (POWO, 2026). The genus name *Schinus* itself derives from the Greek schinos, the mastic tree, applied by resemblance to the aromatic resin. The deeper etymology, whether mulli has a pre-Quechua substrate origin, and how it relates to the ritually charged term mulli, including the possibility of a pre-Quechua substrate origin and its possible relationship with the ritually charged term mullu, the Spondylus shell, remains debated in Andean historical linguistics and is not resolved here.

What matters for the present argument is referential instability. A single vernacular term, applied across thousands of kilometres, came to designate a tree whose modern taxonomic identity is itself unsettled. At the level of the name, “molle” already points to a heterogeneous referent, the first link in the cascade of Figure 1. Etymology is therefore not ornamental in the present paper. The name frames the history of use, and that history becomes pharmacologically difficult to interpret if the botanical referent has shifted across regions, languages and taxonomic treatments.

## 4 Pre-Columbian use: the archaeology of chicha de molle

The best-documented ancient use of molle is chicha de molle, a fermented beverage prepared from the drupes. At the Wari colony of Cerro Baúl in Moquegua, southern Peru, around AD 600–1000, archaeological evidence from a monumental brewery yielded abundant *S. molle* seeds and material evidence consistent with high-status, specialized brewing (Moseley et al., 2005). Botanical identification was established experimentally by Goldstein and Coleman (2004), and DART-MS residue analysis of brewery ceramics subsequently identified molle-specific triterpenoids, hydroxymasticadienoic and (iso) masticadienoic acids, moronic acid, schinol and simiarenol, distinguishing molle chicha from maize chicha (Williams et al., 2019).

Two points are relevant to the present framework. First, molle chicha appears to have been locally produced and poorly suited to long-distance storage or transport. Such locality is not merely social; it also invites chemical variability because the plant material used in different valleys may have reflected different populations, soils, climates and harvest conditions. Second, the triterpenoids used to detect molle archaeologically belong to the same broad chemical repertoire whose variation future chemotype work must examine. Archaeology and phytochemistry therefore read the same molecules, but at different temporal scales.

Beyond documenting antiquity, this record is hypothesis-generating. Large-scale, spatially organized production of chicha de molle implies a tradition that selected and managed particular material, which is what makes it reasonable to expect traditional-use clusters to track chemistry rather than nomenclature—the expectation formalized in prediction P5.

## 5 Ethnomedicinal and pharmacological record: the traditional analgesic/anti-inflammatory signal

Across its range, *S. molle* is repeatedly recorded in Andean folk medicine as an external remedy for musculoskeletal pain and inflammation, especially through leaf decoctions, baths and cataplasms, within regional medicinal-plant traditions documented by market-based and community-based ethnobotanical studies (Macía et al., 2005; Bussmann, 2013; Monigatti et al., 2013). Notably, the Bolivian market survey of Macía et al. (2005) found that musculoskeletal complaints—rheumatism, contusions, sprains and swellings—formed one of the largest categories of recorded use, the indication most relevant here.

Table 4 does not attempt to validate each traditional use. Instead, it separates traditional records, experimental findings and their degree of alignment with the specific signal most relevant to this study: external leaf-based analgesic and anti-inflammatory use.

**TABLE 4:** Documented and experimentally examined uses of Schinus molle, with supporting literature and evidential alignment.

Use	Part/preparation	Evidence type	Ethnopharmacological alignment	Key source(s)
Antirheumatic/musculoskeletal	Leaf; topical preparations	Traditional regional surveys	Aligned — core ethnomedical signal	Macía et al. (2005), Monigatti et al. (2013)
Analgesic; CNS-depressor	Leaf; dichloromethane extract	Traditional + rodent *in vivo*	Aligned — pain signal (non-traditional solvent)	Barrachina et al. (1997)
Analgesic, anti-inflammatory, sedative	Dichloromethane extract	Rodent *in vivo*	Aligned — reinforces the analgesic/anti-inflammatory signal	Taylor et al. (2016)
Anti-inflammatory	Leaf/fruit; extract	Traditional + *in vitro*/*in vivo*	Aligned — inflammation signal (matrix varies)	Bello et al. (1998)
Anti-inflammatory	Extract	*In vitro* + *in vivo*	Aligned — recent anti-inflammatory support	Shawa et al. (2025)
Anti-inflammatory compounds	Fruit; triterpenoids + biflavanone	Isolated compounds	Aligned (mechanistic); not a traditional preparation	Yueqin et al. (2003)
Antioxidant; antiproliferative	Berry essential oil	*In vitro*	Not aligned — laboratory discovery	Bendaoud et al. (2010)
Cytotoxic activity	Extracts	*In vitro*	Not aligned — laboratory discovery	Ruffa et al. (2002)
Chilean seed/fruit essential oil	Seed/fruit oil; GC-MS profile	*In vitro* + *in silico*	Not aligned — bioactivity baseline, not analgesia	Oses et al. (2025)

The convergence is the point. Across regions and time periods, the most repeated traditional signal is one specific indication: external anti-inflammatory, analgesic and antirheumatic use of the leaf. Modern preclinical studies do not clinically validate this use, and often employ non-traditional solvents, plant parts or routes of administration. Nevertheless, they provide partial pharmacological support for the plausibility of this traditional signal (Barrachina et al., 1997; Bello et al., 1998; Yueqin et al., 2003). In this precise and limited sense, the ethnomedical record identifies the observation that future chemotype-aware pharmacology should test.

### 5.1 A structured synthesis of the experimental record

The evidence discussed here is drawn from a structured, illustrative synthesis of the published literature on the pharmacology, chemistry and traditional use of molle, rather than from a systematic review. A systematic review of this literature is planned as a separate study; the synthesis presented here is deliberately structured and selective, assembled to expose a pattern in how the object of study is defined rather than to estimate any effect. The corpus comprises primary pharmacological and phytochemical studies of *Schinus molle* and *S. areira*, together with regional ethnobotanical records, and reviews of the genus used for context.

Two inclusion principles were applied. First, a study had to concern traceable plant material of *S. molle* or *S. areira*—assayed pharmacologically or toxicologically, characterized chemically, or documented in traditional use—so that its object could in principle be located on the axes the ethnopharmacological unit tracks. Second, studies of isolated single constituents assayed independently of the plant material, such as purified alpha-phellandrene, were treated as mechanistic support rather than as evidence about the identity of the object. No quantitative meta-analysis was attempted, and the set is illustrative rather than exhaustive, and Table 5 should not be read as a comprehensive or unbiased representation of the literature.

**TABLE 5:** Reported plant material, preparation and endpoint across representative pharmacological studies of molle.

Study	Species	Origin	Voucher/auth.	Part	Preparation	Chemistry	Endpoint
Bello et al. (1998)	*S. molle* L	Valencia, Spain (naturalized)	Authenticated; no number	Leaves	Distilled oil → DCM extract	None (yield only)	Smooth-muscle antispasmodic
Yueqin et al. (2003)	*S. molle* L	Valencia, Spain (cultivated)	Voucher DF10/01	Fruits	MeOH extract; isolated compounds	Isolated cpds (NMR/MS)	Anti-inflammatory
Ruffa et al. (2002)	*S. molle*	Buenos Aires/Entre Ríos, Argentina (native)	Voucher (Farmacobotánica)	Aerial	Methanol extract	None (screening)	Cytotoxicity (hepatocarcinoma)
Bendaoud et al. (2010)	*S. molle* L	Sfax, Tunisia (naturalized)	Not reported	Berries	Essential oil (steam dist.)	GC-MS	Anticancer; antioxidant
Martins et al. (2014)	*S. molle* L	Évora, Portugal (naturalized)	Voucher (Univ. Évora)	Leaves + fruits	Essential oil	GC-MS; varies by region	Antioxidant; antimicrobial; toxicity
Herrera-Calderon et al. (2022)	*S. molle* L	Ica, Peru (cultivated)	Not reported	Leaves	Essential oil (hydrodist.)	GC-MS	Antioxidant; insecticidal (*in silico*)
Oses et al. (2025)	*S. molle* L	Atacama, Chile (native)	Voucher CONC 125734	Seeds	Essential oil (steam dist.)	GC-MS (α-phellandrene)	Antioxidant; antitumor; toxicity

For a representative set of pharmacological studies we recorded, where reported, the species name as given, the geographic origin of the material, the voucher or authentication status, the plant part, the preparation, the chemical characterization, and the pharmacological endpoint (Table 5). Fields not reported in a source are marked as such, because the absence of a voucher or of a stated provenance is itself informative for the argument developed here.

Read across studies, the label “*Schinus molle*” is attached to materials that differ on every axis the ethnopharmacological unit tracks. The material originates from at least six countries across three continents—Spain, Portugal, Tunisia, Argentina, Peru and Chile—spanning both the native Andean range and naturalized or cultivated European and North-African stands; voucher documentation is present in some studies and absent in others; the plant part ranges from leaves to fruits to seeds to whole aerial material; and the preparation ranges from a dichloromethane extract to a methanol extract to a steam- or hydro-distilled essential oil. Chemical characterization, where performed, itself varies with geographic origin (Martins et al., 2014), and distinct chemotypes are reported—for example, an alpha-phellandrene–dominant oil in Atacama material (Oses et al., 2025). Each study may be sound in its own terms, yet together they do not describe a single pharmacological object. This is the heterogeneity the Mulli Hypothesis identifies, made explicit study by study; it is why a result obtained in one row cannot be assumed to transfer to another under the shared binomial alone.

Two clarifications about the evidential status of this synthesis follow. Table 5 documents heterogeneity of material and reporting; it deliberately does not include effect sizes, sample sizes, doses, replicates or statistical comparisons between materials, and it therefore does not—and is not intended to—demonstrate that the ethnopharmacological unit predicts pharmacological activity better than the species label. It illustrates the problem and motivates the test, which is the role of the predictions in Table 1. Relatedly, the causal chain the Mulli Hypothesis proposes—from taxonomic and chemical heterogeneity to pharmacological irreproducibility—is advanced as a testable hypothesis, not a demonstrated relationship: the evidence assembled here shows that studies differ in name, provenance, part, preparation and chemistry, but not yet that these differences produce different activities, which is precisely what the predictions are designed to establish.

The record is also not uniformly positive, and the conflicts are worth stating concretely rather than merely acknowledging. The anti-inflammatory and analgesic effect is not universal: it appears in leaf and fruit preparations tested for pain and oedema (Barrachina et al., 1997; Yueqin et al., 2003), but is weak or secondary in several essential-oil and aqueous preparations, whose activity is dominated instead by antioxidant and antimicrobial effects. Antimicrobial potency itself varies with geographic origin and chemotype, pronounced in some essential oils and modest in others (Bendaoud et al., 2010; Martins et al., 2014). And the safety signal is internally divided: the same organ yields a non-genotoxic aqueous extract and a genotoxic essential oil (Machado et al., 2024; Duarte et al., 2018). Under the Mulli Hypothesis these are not results to be reconciled as properties of one entity but what one would expect if different objects are being assayed under one name; treating them as contradictions within a single species is precisely the error the framework diagnoses.

### 5.2 The binomial underdetermines the phenotype beyond Schinus

The problem the Mulli Hypothesis identifies for molle is not unique to it. In several well-studied medicinal plants the botanical binomial is known to underdetermine the pharmacological phenotype, because clinically relevant chemical variation exists within the named species (Table 6). These cases are offered not as evidence that the ethnopharmacological unit is correct, but to show that the problem it addresses is general, and that the unit may therefore be applicable beyond a single Andean tree.

**TABLE 6:** Comparative cases in which intraspecific chemical variation makes the botanical binomial an insufficient predictor of the pharmacological phenotype.

Species	Axis of intraspecific chemical variation	Why the binomial underdetermines the phenotype	References
*Cannabis sativa*	THC- vs. CBD-dominant vs. mixed chemotypes (locus B)	Psychoactivity, therapeutic profile and legal status track the chemotype, not the species name	de Meijer et al. (2003)
*Curcuma longa*	Curcuminoid content varies ∼10-fold with cultivar, locality and extraction	The active-compound dose is not specified by the binomial; products differ markedly	Li et al. (2011)
*Hypericum perforatum*	Hyperforin (antidepressant principle) and hypericin vary with genotype, origin and harvest; hyperforin degrades on storage	Two *H. perforatum* extracts can differ in the actual active compound	Carrubba et al. (2021)
*Arnica montana*	Helenalin- vs. dihydrohelenalin-dominant chemotypes by altitude/provenance	Potency and toxicity of the anti-inflammatory sesquiterpene lactones differ by chemotype	Perry et al. (2009)
*Schinus molle*	Provenance, part, preparation and chemotype vary widely (Table 5)	Studies under one name do not describe one pharmacological object	This work (Table 5)

In each case the pattern is the same: a single Latin name is attached to material whose pharmacologically decisive chemistry varies with genotype, provenance, plant part, harvest or preparation, so that a botanical name alone may underdetermine pharmacologically relevant chemical variation; whether an ethnopharmacological-unit classification improves prediction of the pharmacological phenotype remains an empirical question. *Schinus molle* is one instance of a general condition, not an isolated curiosity. Where that condition holds, an analytical unit that makes the relevant covariates explicit—the ethnopharmacological unit proposed here—applies in principle to any of these species.

### 5.3 Identifying and validating chemotypes

Because the chemotype is a defining field of the ethnopharmacological unit, it is worth being explicit about how a chemotype is identified and how far the existing identifications can be trusted. The standard approach profiles the volatile fraction by GC-FID and GC-MS (and the non-volatile fraction by LC-MS where relevant), assembles a matrix of relative constituent abundances across samples, and applies unsupervised multivariate analysis—principal component analysis and hierarchical cluster analysis—to detect groupings, which are then labelled by their dominant markers. For molle this has already been done: a systematic review of the essential-oil literature used hierarchical clustering of relative compositions and recovered two prevalent chemotypes, α-phellandrene- and sabinene-dominant, with a marker defined as the constituent exceeding roughly 15% of the oil (Radice et al., 2026); an earlier study of eleven Brazilian populations resolved four groups by average-linkage clustering, characterized respectively by sabinene, α- and β-pinene, α-cadinol and myrcene (Gomes et al., 2013).

Two cautions follow. First, most chemotype assignments for molle are descriptive—a sample is placed by its dominant compound—rather than validated: they are drawn from independent studies that differ in provenance, plant part, extraction method and analytical platform, so the clusters partly reflect methodological variation as well as biology. Second, a chemotype recovered from pooled literature is not the same as one demonstrated by design. Validating a chemotype requires a common sampling frame, replicated profiling, a clustering solution tested for stability (for example, by PERMANOVA on chemical distance and by resampling), and, ideally, confirmation in an independent collection—precisely the design specified for prediction P1 (Table 1). The chemotype field of an ethnopharmacological unit should therefore carry the same certainty tag as its other fields: it is useful to distinguish three levels explicitly: general chemical-profile variation (differences that may arise from environment, organ, season, drying, storage, extraction or analytical platform); a putative chemotype (a recurrent dominant-compound pattern identified from such data but not yet shown to be stable); and a genetically stable chemotype (a pattern confirmed to persist under replicated and, ideally, common-garden sampling). Differences in dominant compounds should not be read as stable chemotypes by default, and most current assignments for molle are of the first two kinds. Accordingly, a literature-derived, dominant-compound assignment is a reasonable inferred value, whereas a design-validated chemotype is a confirmed one, and the two should not be treated alike.

## 6 Pharmacological implications of the ethnopharmacological unit

A larger pharmacological literature supports the plausibility of the ethnomedical signal, but it also indicates why standardization should precede translation. Barrachina et al. (1997) reported analgesic and central-depressor effects for a dichloromethane extract from *S. molle* leaves in rodents. Bello et al. (1998) reported *in vitro* pharmacological activity for the same extraction context, and Yueqin et al. (2003) isolated triterpenoids and a biflavanone with anti-inflammatory activity from fruits. More recent studies on *S. molle* extracts have further examined analgesic or anti-inflammatory activity and should be interpreted within the same cautionary frame (Taylor et al., 2016; Shawa et al., 2025). These studies are not clinical validation, and they do not reproduce a traditional topical decoction. Nevertheless, they indicate that biologically active fractions exist in the species complex and that the anti-inflammatory signal is not merely rhetorical or folkloric. Reviews of the genus as a whole indicate that this chemical and pharmacological diversity is not restricted to *S. molle* but characterizes *Schinus* more broadly (El-Nashar et al., 2022).

A mechanistic bridge may come from monoterpene pharmacology, particularly alpha-phellandrene, a recurrent constituent of several *S. molle* essential oils (Herrera-Calderon et al., 2022). Isolated alpha-phellandrene has shown antinociceptive and anti-inflammatory effects in rodent models (Lima et al., 2012; Siqueira et al., 2016). These mechanisms do not prove that an *S. molle* preparation acts in the same way. They provide, more modestly, a pharmacological rationale for asking whether chemotypes enriched in particular monoterpenes display more consistent anti-inflammatory or antinociceptive profiles.

Recent work in Chile makes the translational question more local and more concrete. Oses et al. (2025) characterized an essential oil from Chilean *S. molle* material and reported a profile dominated by alpha-phellandrene, beta-myrcene, limonene and beta-phellandrene, together with antioxidant, antimicrobial, antiproliferative and toxicity assays. That study did not test pain, evaluate human participants or propose a clinical trial. Its value for the present framework is different: it indicates that Chilean material can be chemically characterized and biologically screened, providing a platform on which identity-confirmed, chemotype-aware analgesic work could be built.

The pharmacological argument therefore has three layers. The first is ethnomedical: external leaf use for musculoskeletal pain and inflammation. The second is preclinical: extracts and isolated compounds show anti-inflammatory or antinociceptive activity in non-human systems. The third is chemical: different organs and populations contain different mixtures of volatile and non-volatile constituents. The Mulli Hypothesis states that these layers should not be collapsed. A future candidate cannot be “*S. molle*” in the abstract. It must be a defined ethnopharmacological unit with a voucher specimen, provenance, plant part, preparation, chemical profile and bioactivity range.

This framing also prevents premature clinical enthusiasm. A topical product for musculoskeletal pain would require, at minimum, identity-confirmed plant material, a chemically specified extract or oil, stability data, dermal safety, a placebo-matched vehicle, and preclinical evidence that the chosen matrix is relevant to the traditional indication. Direct safety and toxicity data for *S. molle* preparations remain limited (Carrasco-Solano et al., 2023). More pharmacology is therefore not a call to rush toward a trial; it is a call to design the preclinical bridge properly.

For ethnopharmacology, the pharmacological question should be formulated in a culturally precise way. A generic question such as whether *S. molle* is analgesic is too broad and too poorly anchored. A better question is whether a defined Andean ethnopharmacological unit, for example, a voucher-confirmed leaf material from a specified provenance, prepared in a way that approximates topical traditional use and chemically profiled before testing, would produce a reproducible anti-inflammatory or antinociceptive phenotype. This phrasing preserves the traditional observation while making it experimentally tractable. It also prevents common translational errors: replacing the traditional plant part with a convenient oil, replacing a topical preparation with an oral dose, or treating a market name as a taxonomic identification. In this sense, pharmacology becomes a test of a biocultural object, not merely a screen of a plant extract.

The same logic applies to outcome selection. The traditional cluster points most strongly to musculoskeletal pain, swelling and rheumatic complaints. Future preclinical studies should therefore prioritize models relevant to inflammatory hyperalgesia, mechanical sensitivity and local tissue inflammation before expanding to distant outcomes such as tumour cytotoxicity or vector control. Such prioritization does not deny the broader bioactivity literature; it simply prevents the historical signal from being diluted by every possible biological assay. A standardizable candidate should emerge from the intersection of traditional relevance, chemical reproducibility, biological plausibility and safety, not from activity alone.

*Schinus molle* has also shown antimicrobial, antioxidant, insecticidal, anticancer and antiviral activities. We foreground the analgesic and anti-inflammatory signal not because the others are unimportant, but because it is the one activity that converges between the traditional record and the experimental literature; convergence of this kind is what the framework treats as the strongest lead. We present it as a priority for testing rather than as preliminary validation: the independence and number of the ethnobotanical sources, the comparability of the traditional preparations, and the correspondence between traditional and experimental routes of administration remain insufficiently documented, so external leaf-based analgesic use is best regarded as the strongest candidate for focused future study rather than as an activity already supported.

Mechanistically, the reported activities map onto major chemical classes rather than onto any single compound. The monoterpenes that dominate the essential oil—α- and β-phellandrene, sabinene and limonene—are most associated with the antinociceptive and anti-inflammatory signal, α-phellandrene having shown antinociceptive activity in rodent models through several convergent pathways—opioidergic, glutamatergic, nitrergic, cholinergic and adrenergic—and anti-inflammatory activity through suppression of pro-inflammatory cytokines (IL-6, TNF-α) and modulation of transient-receptor-potential channels (Lima et al., 2012; Siqueira et al., 2016); the triterpenoids and biflavanone isolated from the fruit act on eicosanoid pathways, reducing phospholipase-A2- and leukotriene-mediated inflammation (Yueqin et al., 2003); and the polyphenols and tannins of aqueous preparations account for much of the antioxidant capacity. Because these classes are partitioned differently by provenance, chemotype and preparation, we hypothesize that the mechanisms engaged by a given preparation may depend on the specific combination of provenance, chemical profile, plant part and preparation represented by an ethnopharmacological unit, rather than being predictable from the species label alone. This mechanistic evidence, moreover, comes largely from isolated constituents rather than whole preparations, and is best read as a class-level rationale: which pathways a given remedy actually engages depends on which constituents its preparation contains.

## 7 Taxonomy and the molle/areira problem

The taxonomic instability of molle provides a concrete example of why the Latin binomial alone is an insufficient ethnopharmacological unit. Linnaeus described both *S. molle* and *S. areira*; De Candolle later subordinated the latter as *S. molle* var. areira, a treatment followed by Barkley (Barkley, 1944; 1957). The two entities were subsequently separated on leaflet characters whose limits overlap, and a recent study lectotypified *S. areira* and redefined its diagnostic characters, treating it as a distinct species (Zapater et al., 2018). Plants of the World Online currently accepts *S. areira* L. (POWO, 2026).

This matters because the two species share part of their range and were long regarded as synonyms. Reports published under one name may therefore concern the other. Zapater et al. (2018) note explicitly that earlier records attributed to *S. molle* probably correspond to *S. areira*. If so, a substantial fraction of the “*S. molle*” pharmacological literature may rest on more than one biological entity. Reported variability would then be partly a species-identity artefact and partly a genuine chemotype signal. Disentangling the two with vouchered, identity-confirmed material is a precondition for interpretation. The instability is not confined to the pharmacological literature: recent Argentine ethnobotanical surveys record the tree specifically as *S. areira*—for respiratory complaints in the Calchaquí Valleys (Fabbroni et al., 2022) and as the “pimiento” of Huarpe communities in San Luis (Moglia et al., 2023), so that even the ethnomedical record is distributed across both binomials. Table 7 summarizes the taxonomic names relevant to the molle/areira problem and should appear here.

**TABLE 7:** Taxonomic names relevant to the molle/areira problem.

Name	Current or historical treatment	Relevance
*Schinus molle* L	Accepted species	Principal name used in pharmacological literature
*Schinus areira* L	Accepted species in POWO (Zapater et al., 2018; POWO, 2026); historically conflated with *S. molle*	Possible source of identity confusion
*S. molle* var. areira (L.) DC.	Historical varietal treatment [De Candolle]	Accounts for the long-standing synonymy in the literature
Molle Mill. (1754)	Generic synonym (POWO, 2026)	Reflects pre-Linnaean naming history

**FIGURE 5:**
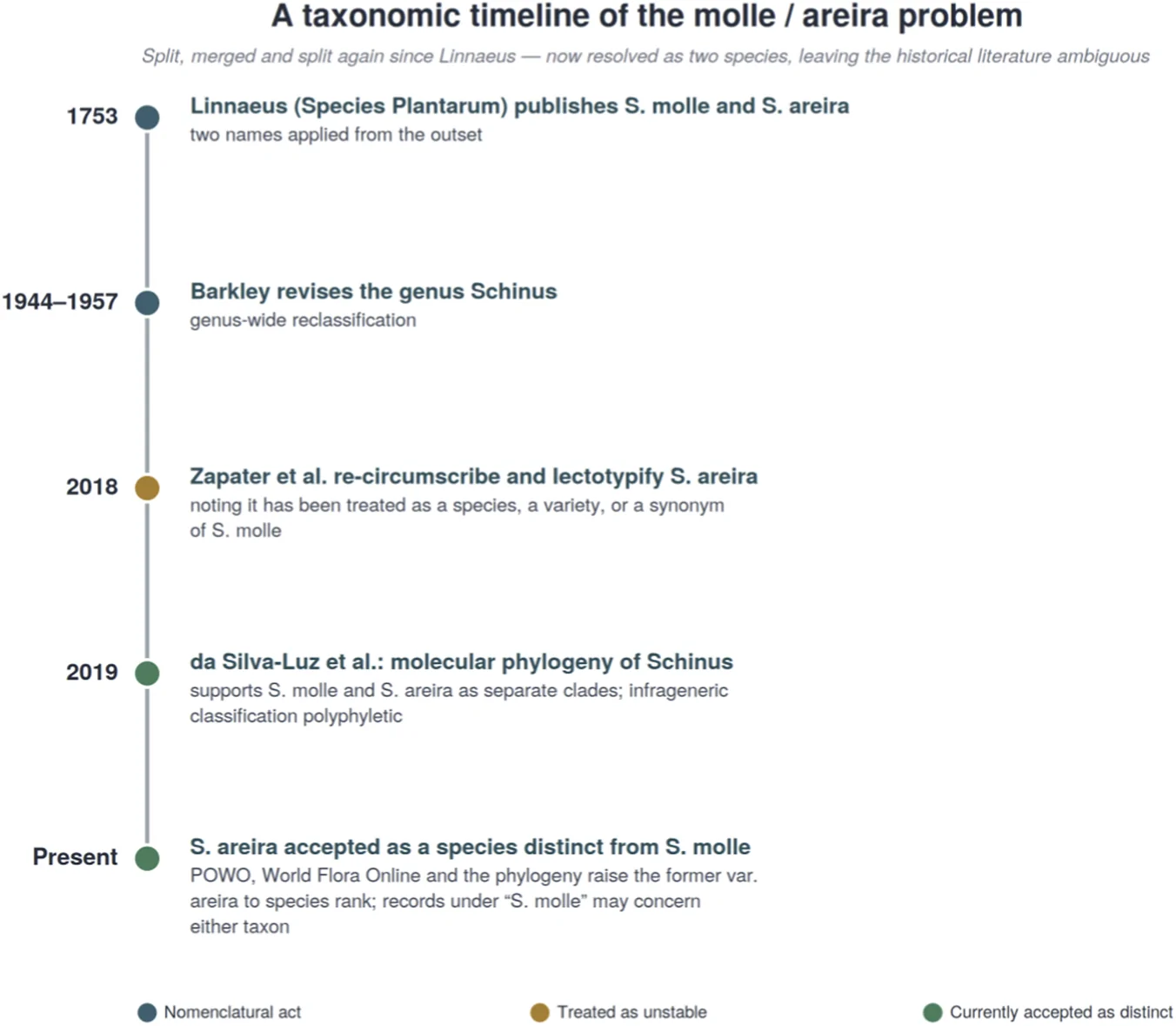
A taxonomic timeline of the molle/areira problem. The identity of the plant has shifted repeatedly between one and two species since Linnaeus; current databases and the molecular phylogeny now treat *Schinus areira* as distinct from *Schinus molle*, so that historical records under “*Schinus molle*” cannot be assumed to refer to a single currently accepted taxon.

The instability described so far is nomenclatural and historical, but it is neither confined to the past nor dissolved by modern data, and it is worth asking what molecular and systematic evidence adds. Three lines are relevant. First, the most comprehensive molecular phylogeny of the genus — 44 taxa and eleven nuclear and plastid regions—recovers *Schinus* as monophyletic but finds its traditional infrageneric classification to be polyphyletic, and states that species delimitation within the genus remains a challenge (da Silva-Luz et al., 2019). Second, molecular systematics reframes rather than removes the molle/areira question: current taxonomic databases and the phylogeny now agree in treating *S. areira* as a species distinct from *S. molle*, raising the former *S. molle* var. *areira* to species rank (Plants of the World Online, 2026; da Silva-Luz et al., 2019; Figure 5). The instability is thus historical rather than a present disagreement between databases—but because much of the pharmacological literature was published under ‘*S. molle*’ during the long period when the two were merged, that literature cannot be assumed retrospectively to refer to a single currently accepted taxon.

Third, population-genetic analysis of S. molle using amplified fragment length polymorphism (AFLP) markers reports low genetic diversity but clear genetic structure among populations (Lemos et al., 2015), indicating genetic differentiation among populations. Taken together, the molecular evidence does not remove the problem the Mulli Hypothesis invokes but sharpens it: by separating S. areira from S. molle it indicates that one historical name has been applied to more than one currently recognized species, exactly the hidden heterogeneity the ethnopharmacological unit is designed to make explicit.

This bears directly on how much of the chemical variation is heritable rather than environmental. The population structure detected genetically (Lemos et al., 2015) is consistent with a heritable component, although genetic differentiation among populations does not by itself demonstrate heritability of chemotype; establishing that would require common-garden, family or genotype-based testing. The dependence of essential-oil composition on geographic origin, altitude, organ and harvest season (Gomes et al., 2013; Radice et al., 2026) likewise indicates that an environmental component exists. Their relative contributions have not been established for molle and cannot be separated from observational data alone; disentangling them is exactly what the common-garden design of prediction P3 is intended to do. Until that is done, neither genotype nor environment should be assumed to dominate—a caution the ethnopharmacological unit encodes by recording provenance and chemotype as separate fields rather than collapsing them.

## 8 Where traditional knowledge and pharmacology diverge

A credible study must resist a confirmatory reading. Traditional knowledge and modern pharmacology do not map onto each other cleanly, and the mismatches are as informative as the agreements. Much of the contemporary literature concerns activities with little basis in the Andean analgesic tradition: broad antimicrobial and antifungal effects (Martins et al., 2014), insecticidal and repellent activity against *Triatoma infestans* and other arthropods (Ferrero et al., 2006; Achimón et al., 2022), antiproliferative or cytotoxic effects on tumour cell lines (Ruffa et al., 2002; Bendaoud et al., 2010; Oses et al., 2025), and other laboratory observations. These are laboratory discoveries rather than outcomes directly anticipated by the core ethnomedical record. The convergence between historical knowledge and pharmacology applies to a specific indication, not to the species’ full pharmacological range.

Conversely, several recorded uses, diuretic, gynaecological, purgative and ritual or veterinary applications, have weak or absent modern experimental support. A further mismatch concerns preparation: the tradition emphasizes topical leaf decoctions, whereas much bioactivity work tests fruit essential oil, dichloromethane extracts or isolated compounds. These matrices may be useful experimentally, but they may not represent the traditional remedy. The difference is not a minor methodological issue. In ethnopharmacology, plant part and preparation are part of the intervention. A fruit essential oil and a leaf decoction should not be interpreted as equivalent simply because both come from a plant called molle.

These are not interchangeable chemical samples. Steam or hydro-distillation captures the volatile monoterpene fraction (α-phellandrene, sabinene and related terpenes); organic-solvent extraction with dichloromethane or methanol recovers semipolar constituents and triterpenoids; and aqueous decoctions and infusions extract polyphenols, tannins and proanthocyanidins. A preparation therefore selects a chemical subset, so that a traditional topical leaf decoction and a laboratory fruit essential oil may share a species and still contain largely different compounds—which is why preparation is a mandatory field of the ethnopharmacological unit.

Recognizing divergence strengthens, rather than weakens, the central claim. Agreement is partial because “molle” pools heterogeneous entities, parts, preparations and local meanings. The ethnopharmacological unit is a way to make those differences explicit rather than hiding them behind a single species name.

This divergence is itself a prioritization tool. Activities that converge across the traditional and experimental records—external leaf-based analgesia above all—are the strongest candidates for phytopharmaceutical development, whereas laboratory activities with no traditional anchor warrant more cautious interpretation before resources are committed.

## 9 Discussion

Read as a whole, the record of *S. molle* is of a heterogeneous, regionally diversified entity. Three implications discipline modern research. First, biological identity is not a preliminary methodological step but part of the therapeutic hypothesis itself: every pharmacological study should rest on voucher-confirmed material and a declared taxonomic concept (Bennett and Balick, 2014). Second, provenance and chemotype are explanatory variables, not nuisances: locality, ecology, plant organ and preparation are part of the scientific object. Third, the ethnomedical anchor should be specific: the most coherent translational target is external leaf-related analgesia and anti-inflammation, tempered by the divergences described above.

**FIGURE 6:**
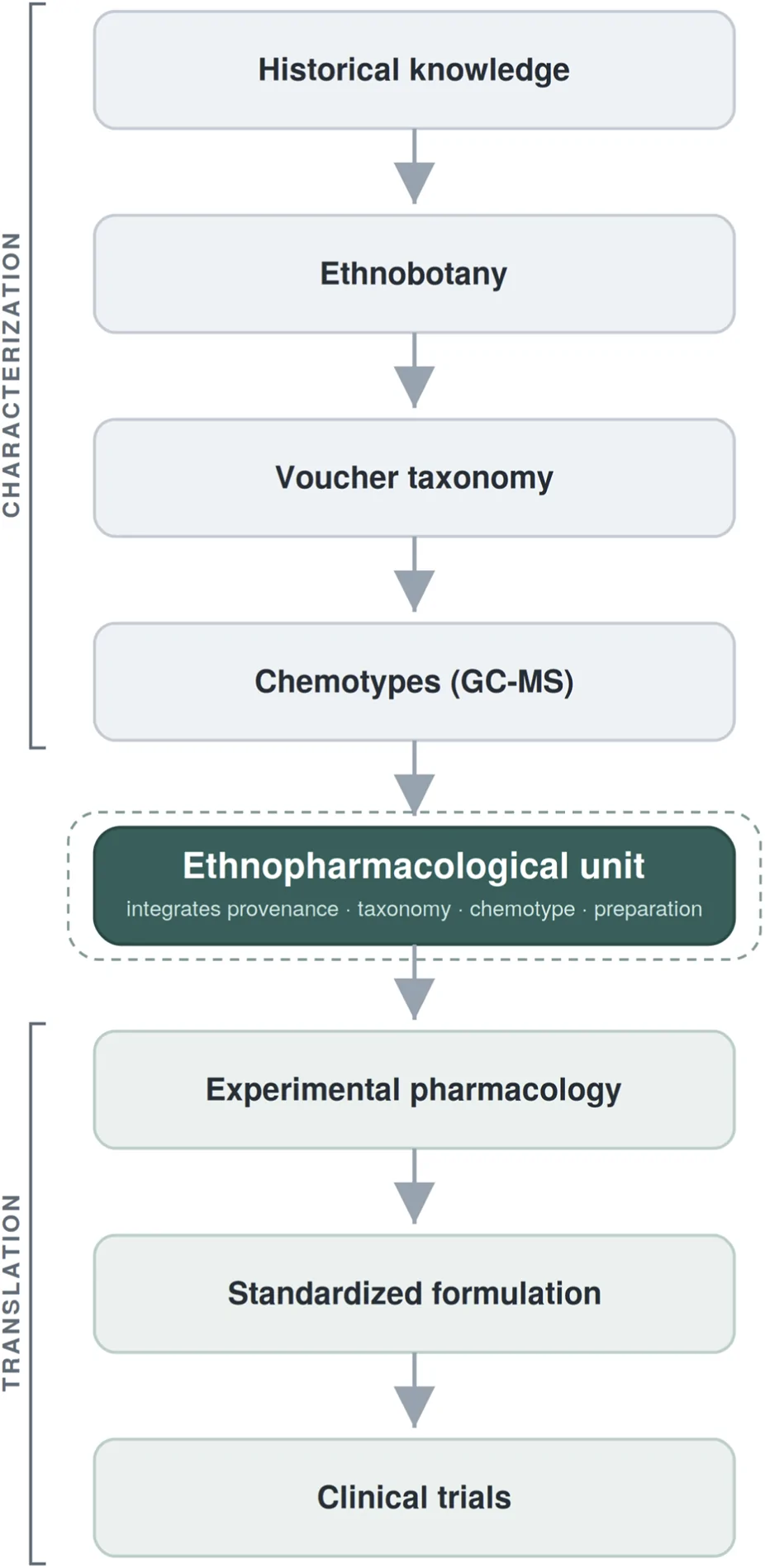
A research roadmap for the ethnopharmacological unit. Historically and ethnobotanically defined leads are resolved into voucher-confirmed taxa and GC-MS chemotypes; these layers are integrated by the ethnopharmacological unit, which then structures experimental pharmacology, standardized formulation and clinical evaluation.

Throughout, we speak of a framework for future standardization and a standardizable phytopharmaceutical candidate, not an accomplished product. No demonstrated chemotype, validated formulation or clinical evidence yet exists. The purpose of this study is to establish why the historical, ethnobotanical, taxonomic and phytochemical evidence make the Mulli Hypothesis a warranted and testable proposal. Its contribution is conceptual: the hypothesis, its falsifiable predictions, and the ethnopharmacological unit. As a conceptual contribution, the framework is intentionally prospective; its value lies in making its assumptions explicit, operational and open to falsification. This staged programme—from historical and ethnobotanical characterization, through voucher taxonomy and chemotype resolution, to the ethnopharmacological unit and its translational evaluation—is summarized in Figure 6.

For Chile, the framework is strategically useful. Northern Chile lies within the biocultural and ecological horizon of the molle/areira problem, and recent Chilean chemical work indicates that local material can be profiled with contemporary analytical tools (Oses et al., 2025). A responsible research programme would not begin with a clinical claim. It would begin with populations, vouchers, local knowledge, chemotypes, traditional preparations and preclinical assays aligned with the intended indication.

These principles translate into concrete methodological recommendations for future work on molle and comparable taxa: voucher specimens should be collected and taxonomically confirmed before bioactivity testing; plant part, preparation, provenance and local indication should be reported explicitly; chemotypes should be compared rather than pooled under a single species label; laboratory matrices should be aligned with the traditional preparation; and benefit-sharing should be planned with local knowledge holders. Together these steps operationalize the ethnopharmacological unit as a practical sampling and reporting standard.

The heterogeneity of the object is not the only possible source of the inconsistent pharmacological record, and the framework does not claim that it is. Reported activity also varies with methodological factors independent of the plant: differences in assay systems, cell lines and animal models; in extraction solvent, dose and vehicle; in season of harvest, plant age, cultivation and storage; in analytical platform; and in laboratory-to-laboratory variability; and in the completeness of reporting, compounded by the tendency of positive results to be published more readily than negative ones. These sources are real and must be controlled. Our claim is narrower and complementary: that object-level heterogeneity is an additional and frequently unexamined source of variability, and that it can be separated from the methodological sources by the procedure in Figure 2 rather than conflated with them.

### 9.1 Safety, toxicology and regulatory translation

The safety of molle has been examined, but—as with its efficacy—the available data describe several different objects rather than one. Acute oral administration of a leaf aqueous extract produced no acute toxicity and no genotoxicity in the comet and micronucleus assays up to 2000 mg/kg (Machado et al., 2024), and subchronic dietary exposure to ethanolic leaf and fruit extracts caused no histopathological alterations in mice (Bras et al., 2010). Acute dermal exposure to ethanolic and hexanic leaf extracts caused only slight, reversible skin irritation, consistent with the traditional topical use (Bras et al., 2011); notably, *S. molle* lacks the urushiol-type contact allergens that make some other Anacardiaceae dermatologically hazardous, although oxidized monoterpenes in its essential oil remain a minor irritant risk. Against this generally reassuring picture, the leaf essential oil has shown concentration-dependent genotoxicity in macrophages (Duarte et al., 2018), and cytotoxicity through apoptosis in tumour cell lines (Oses et al., 2025).

The internal contrast in these data is the point. A leaf aqueous extract is non-genotoxic, whereas a leaf essential oil from comparable material is genotoxic at higher concentrations: same species, same organ, opposite safety signal, because the preparation differs. This cross-study contrast is consistent with safety being a property of the ethnopharmacological unit rather than of the binomial; because these studies also differ in assay, dose, route, laboratory and material, however, the comparison motivates this hypothesis rather than establishing it. A claim that “S. molle is safe” is not well formed until the unit—provenance, part, preparation and chemotype—is specified. This also identifies real gaps. The safety domains examined so far are uneven: acute and subchronic oral toxicity, acute dermal toxicity and *in vitro* genotoxicity have been assessed, whereas chronic and reproductive toxicity, dermal sensitization, and long-term or organ-specific studies remain largely unexamined—and none of the existing work is stratified by chemotype. The ethnopharmacological unit provides the natural stratification for closing these gaps.

These considerations connect the framework to the regulatory pathway for botanical medicines. Frameworks such as the U.S. FDA botanical drug guidance and the European Medicines Agency’s provisions for herbal medicinal products require a defined botanical article and demonstrable batch-to-batch consistency, and good agricultural and collection practice governs the starting material. Each presupposes that the object has been defined; none supplies the definition. The ethnopharmacological unit is intended to sit precisely at that upstream point: by fixing identity, provenance, preparation and chemotype as recorded, confidence-rated fields, it converts “*S. molle* material” into a specified article to which quality specifications and consistency criteria can be attached. Framed this way, the unit is a pre-regulatory scaffold as much as a research tool, and a responsible translational programme would establish the unit before, not after, a clinical claim is contemplated.

Limitations are clear. The synthesis is interpretive; finer historical-linguistic questions require specialist primary-source scholarship; and the Mulli Hypothesis and ethnopharmacological unit are frameworks to be tested, not demonstrated results. Because the proposed model requires future chemical, genetic and pharmacological testing, its predictive value remains open. That openness is not a weakness of the framework but a condition of its usefulness.

## 10 Conclusion

From the mulli of the Andes to the modern laboratory, *S. molle* is best understood not as a single, self-evident pharmacological object, but as a heterogeneous biocultural, taxonomic and chemical entity. The traditional record preserves a specific analgesic and anti-inflammatory signal, but that signal can be interpreted responsibly only if the material under study is defined with botanical, geographic, chemical and preparative precision. Treating heterogeneity as the object of study, through the Mulli Hypothesis, its falsifiable predictions and the ethnopharmacological unit, is therefore proposed as a step toward any future framework for standardization. The value of this framework now depends on its explicit testing in identity-confirmed, chemotype-aware and traditionally anchored pharmacological studies. The ethnopharmacological unit is not intended to replace taxonomy, but to complement it whenever pharmacological reproducibility depends on historical, cultural and chemical heterogeneity. Looking ahead, the same unit could structure botanical drug discovery, precision phytotherapy and regulatory science: by defining the object before the assay, it could offer a route from a heterogeneous ethnobotanical record toward a more reproducible, quality-controllable phytopharmaceutical.

## Data Availability

The raw data supporting the conclusions of this article will be made available by the authors, without undue reservation.
